# Fermentation improves antioxidant capacity and γ-aminobutyric acid content of Ganmai Dazao Decoction by lactic acid bacteria

**DOI:** 10.3389/fmicb.2023.1274353

**Published:** 2023-11-02

**Authors:** Linya Wei, Yiming Li, Zina Hao, Zhenjie Zheng, Huixin Yang, Suixin Xu, Shihan Li, Lili Zhang, Yunhe Xu

**Affiliations:** ^1^Department of Food and Health, Jinzhou Medical University, Jinzhou, China; ^2^Comparative Molecular Biosciences Graduate Program, University of Minnesota, Minneapolis, MN, United States; ^3^Innovation Center of Meat Processing and Quality Control Technology of Liaoning Province, Jinzhou, China

**Keywords:** Ganmai Dazao Decoction, GABA, lactic acid bacterial fermentation, antioxidant effects, non-targeted metabolomics

## Abstract

**Introduction:**

Ganmai Dazao Decoction is a traditional Chinese recipe, and is composed of licorice, floating wheat, and jujube.

**Methods:**

Effects of lactic acid bacteria fermentation on the physicochemical properties, antioxidant activity, and γ-aminobutyric acid of Ganmai Dazao Decoction were studied. The changes of small and medium molecules in Ganmai Dazao Decoction before and after fermentation were determined by LC–MS non-targeted metabolomics.

**Results:**

The results showed that the contents of lactic acid, citric acid, acetic acid, and total phenol content increased significantly, DPPH free radical clearance and hydroxyl free radical clearance were significantly increased. γ-aminobutyric acid content was 12.06% higher after fermentation than before fermentation. A total of 553 differential metabolites were detected and identified from the Ganmai Dazao Decoction before and after fermentation by partial least squares discrimination and VIP analysis.

**Discussion:**

Among the top 30 differential metabolites with VIP values, the content of five functional substances increased significantly. Our results showed that lactic acid bacteria fermentation of Ganmai Dazao Decoction improves its antioxidant effects and that fermentation of Ganmai Dazao Decoction with lactic acid bacteria is an innovative approach that improves the health-promoting ingredients of Ganmai Dazao Decoction.

## 1. Introduction

Ganmai Dazao Decoction (GMDZD) is a traditional Chinese recipe with a history of more than 2000 years, and is composed of licorice, floating wheat, and jujube. Modern research shows that GMDZD has sedative, hypnotic, anti-convulsive, anti-depression, and other effects; studies have shown that GMDZD is used to nourish the heart, tranquilize the mind, and alleviate pain and climacteric syndrome (Feng et al., 2016). It is highly effective in treating emotional instability caused by premenstrual syndrome (PMS) in sensitive young women (Shiota et al., 2021). The GMDZD interferes with the balance of gut microbiota and their corresponding metabolites, and plays an anti-Alzheimer’s disease role (Cui et al., 2023). Recent studies have indicated that jujube has extensive pharmacological activities in the nervous system and in anti-oxidation (Chen and Tsim, 2020). Jujube contains γ-aminobutyric acid (GABA; Pu et al., 2019), which is a non-proteinaceous amino acid. It is considered to be the main inhibitory neurotransmitter in the central nervous system (CNS) of vertebrates, playing a key role in maintaining mental health (Pu et al., 2019). Typically, some Lactic acid bacteria (LAB) produces GABA (Men et al., 2019).

LAB fermentation is a simple and valuable technique that improves the bioactive ingredients, functional and sensory properties, and bioavailability of foods for the benefit of human health (Septembre-Malaterre et al., 2018). During fermentation, LAB and their metabolites break down the original ingredients of the food, release bioactive compounds, or synthesize new compounds, which can have beneficial effects on the sensory characteristics and nutritional and functional properties of the food products (Lizardo et al., 2020). Thus they have shown various wide applications in the food industry, such as improving the flavor of fermented foods, increasing food nutrition, reducing harmful substances, and extending shelf life. They can also act as probiotics to promote good health (Wang et al., 2021). Microbial fermentation can provide food with a unique flavor, increase its nutritional value, and enhance its functional characteristics (Xiao et al., 2021). Fermented foods promote nutrient synthesis, prevent cancer and gastrointestinal diseases, and promote anti-allergic reactions (Tamang et al., 2016).

LC–MS is the most widely used technique in metabolomics to analyze unknown metabolites and is often used to evaluate secondary metabolites, such as flavonoids, saponins, alkaloids, phospholipids, and polyamines. Metabolomics of fermented foods enables the prediction of the sensory and nutritional quality of the final products and the observation of their metabolic changes (Utpott et al., 2022). To investigate the influence of LAB fermentation on antioxidant activity, GABA, and small molecule substances in GMDZD, this study used high-performance liquid chromatography (HPLC) and non-targeted metabolomics liquid chromatography-mass spectrometry. This study provides a new basis for the function, nutrition, and healthcare of LAB fermentation GMDZD. It lays a theoretical foundation for further study on the activity and industrial production of fermented GMDZD. It provides new ideas for the development of traditional recipes in the field of nutritional food and functional food.

## 2. Materials and methods

### 2.1. Activation of lactic acid bacteria

Two commercial LAB, YQ336 and YM313, were provided by Microbiology Laboratory, Jinzhou Medical University (Jinzhou, China) and survive in MRS broth with glycerol (25%, v/v). Each LAB is inoculated with 5% and cultured at 37°C for 24 h.

### 2.2. Reagents and standards

The reagents and solvents used are analytical grade or HPLC grade. Organic acid standards, GABA, rutin, gallic acid, and DPPH were obtained from Qingdao Hailan Experimental Equipment Co., Ltd. Acetonitrile, methanol, 4-dimethylaminoazobenzene-4-sulfonyl chloride (Shanghai, China) were used for HPLC.

### 2.3. Preparation of Ganmai Dazao Decoction and fermentation

Licorice, jujube, and floating wheat were purchased from People’s Street Pharmacy in Jinzhou City. This included ripe and whole licorice, floating wheat, and jujube, which were washed with running water to remove dirt and dust. The preparation method of GMDZD is to mix crushed jujube, licorice, and floating wheat with potable water and soak them for 1.5 h. This is followed by a soak in a 90°C water bath for 1 h. before filtering with cotton and nylon cloth. Before fermentation, GMDZD was sterilized at 105° C for 10 min. 5% (v/v) inoculum was added to the sterilized GMDZD, half of which was YM313 and half YQ336; this was cultured at 35° C for 14 h. The physicochemical properties, total phenol content (TPC) and total flavone content (TFC), antioxidant activity, and GABA were measured at 0, 5, 10, and 14 h, respectively. LC–MS samples were taken at 0 and 14 h of fermentation, 0 h as Control and 14 h as treated. All fermentations were performed independently in triplicates.

### 2.4. Determination of physicochemical properties

GMDZD (1 mL) serial dilutions were made in test tubes containing 9 mL of distilled water. Then, 1 mL of diluted GMDZD was inoculated onto petri dishes for plate counting. The colony counts were obtained after culture at 37°C for 48 h. The results are expressed as log CFU/mL of GMDZD. The pH was determined using a digital pH meter. The total and reducing sugars were analyzed as glucose equivalents using the 3, 5-dinitrosalicylic acid method. The titratable acidity was determined by titration with 0.1 M NaOH. HPLC system was used to determine organic acids (Shan Dong, China). The mobile phases were phosphoric acid (pH 2.0) and acetonitrile (98:2). Samples were centrifuged (3,000 × g, 5 min, 4°C) to obtain supernatants and filtered through 0.45 μm organic membranes filters into HPLC sample vials. Standard stock solutions were prepared by distilled water and stored in dark bottles at 4°C. The HPLC results were qualitatively analyzed by peak retention time and quantified by peak area using the external standard method (Li et al., 2021).

### 2.5. Total phenol content and total flavone content

TPC were determined according to a previously reported method (Sun et al., 2022). The content of total polyphenol was determined by the Fulin polyphenol method. All samples were centrifuged at 3000 × g for 5 min, and the supernatant was collected for measurement. Folin–Ciocalteu reagent (3 mL) and 6 mL of 12% Na_2_CO_3_ were added to 1 mL of the supernatant with 15 mL of distilled water. The samples were then incubated for 2 h. in the dark. The absorbance was recorded at 760 nm, and the results were expressed as gallic acid (μg). TFC were determined by the colorimetric method. Firstly, 1 mL 50% (w/v) NaNO_2_ was added to 5 mL of the supernatant. After 6 min, added 1.5 mL 10% (w/v) Al (NO3)_3_ and let the sample react for 6 min. Then 4.0 mL of 4% (w/v) NaOH was added to the mixture and the sample was allowed to react for 15 min. Finally, the absorbance was determined at 510 nm, and the results were expressed as mg rutin equivalents per milliliter.

### 2.6. Determination of antioxidant activities

The DPPH radical scavenging activity was slightly revised from previous reports (Ramirez et al., 2015). Briefly, 2 mL of the sample was added to 2 mL of an absolute ethanol solution of DPPH. The sample was measured after reaction for 30 min. Results were expressed as the percent DPPH radical inhibition. The determination of hydroxyl free radical clearance was as follows: 1 mL 9 mmol/L FeSO_4_ solution, 1 mL 9 mmol/L salicylic acid-ethanol solution, and 1 mL sample solution, and then mixed with 1 mL 8.8 mmol/L H_2_O_2_ solution. After a water bath at 37°C for 30 min, the absorbance was measured at 510 nm. The results are expressed as the percentage of hydroxyl radical inhibition.

### 2.7. **γ**-aminobutyric acid

The mobile phase was acetonitrile: sodium acetate trihydrate solution (35:65). Samples were centrifuged (3,000 × g, 5 min, 4°C) to obtain supernatants and filtered through a 0.45 μm hydrophilic membranes filter into HPLC sample vials. 1 mL was mixed with 0.2 mL sodium bicarbonate solution, and 0.4 mL 4-dimethylamino-azobenzene-4-sulfonyl chloride was added for derivatization. The flow rate of 1 mL/min. Absorbance was measured at 436 nm. Calibration curves were obtained based on 5 levels (0.5, 1.0, 1.5, 2.0, and 2.5 mg/mL) of GABA standard.

### 2.8. Untargeted metabolomics study by LC-tandem MS (MS/MS) technique

For LC–MS, 100 μL treated (GMDZD after 14 h of fermentation) and Control (GMDZD) sample solutions were obtained, and the metabolites were extracted using a 400 μL methanol:water (4:1, v/v) solution with 0.02 mg/mL L-2-chlorophenylalanin as the internal standard. As a part of the system conditioning and quality control process, a pooled quality control sample (QC) was prepared by mixing equal volumes of all samples (all samples were stored at 4°C). Chromatographic separation of metabolites was performed on a Thermo UHPLC system equipped with an ACQUITY UPLC HSS T3 (100 mm × 2.1 mm i.d, 1.8 μm; Waters, Milford, United States). The column temperature was maintained at 40°C, and sample injection volume was 2 μL, and the flow rate was set to 0.4 mL/min (Li et al., 2023). Mass spectrometric data were collected using a Thermo UHPLC-Q Exactive HF-X Mass Spectrometer. This Mass Spectrometer was equipped with an electrospray ionization (ESI) source operating in either the positive ion mode.

The raw data were imported into Progenesis QI 2.3 (Waters Corporation, Milford, United States) for peak detection and alignment. The preprocessing results generated a data matrix that consisted of the retention time (RT), mass-to-charge ratio (m/z) values, and peak intensity. At least 80% of the detected metabolic features in any set of samples were retained and normalized by sum. Metabolic features with a relative standard deviation (RSD) of QC > 30% were discarded. Multivariate statistical analysis was performed using the ropls (Version1.6.2)^1^ R package from Bioconductor on the Majorbio Cloud Platform.^2^ Principal component analysis (PCA) was applied using an unsupervised method to obtain an overview of the metabolic data, and general clustering, trends, and outliers were visualized (Li et al., 2023). Orthogonal partial least squares discriminate analysis (OPLS-DA) was performed to determine global metabolic differences between groups. PCA, cluster analysis, and other analyses were performed on the data using Version 1.0.0. VIP and differential metabolite analyses were performed using Version 1.6.2. MS and MS/MS information was matched to the metabolic databases: Human metabolome database (HMDB) and Metlin database.

### 2.9. Statistical analysis

Results are expressed as the mean ± standard deviation of three independent experiments. Analysis of variance (ANOVA) was performed using SPSS 22 (SPSS, Inc., Chicago, IL, United States), with *p* < 0.05 indicating a statistically significant difference.

## 3. Results and discussion

### 3.1. Physicochemical properties during fermentation

**Figure 1:**
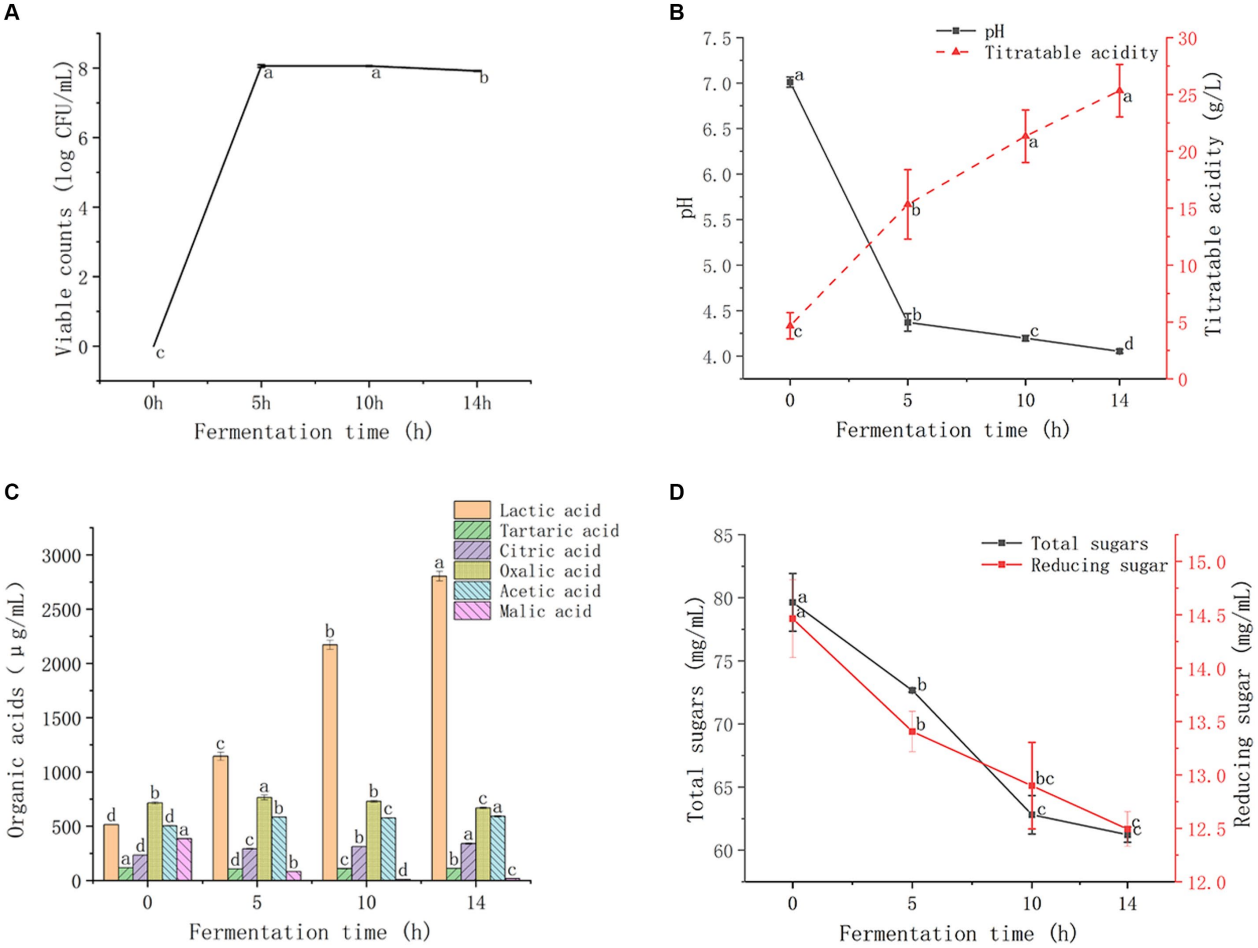
Changes in GMDZD during 14  h of fermentation. The change of viable counts **(A)**, titratable acidity and pH **(B)**,lactic, tartaric, citric, oxalic, acetic, and malic acids **(C)**, and total sugars and reducing sugar **(D)**. Data are expressed as average (*n*  = 3) ± standard deviation.

The viable counts of LAB in GMDZD during the fermentation period are shown in Figure 1A. Owing to sufficient nutrients and suitable growth conditions, LAB grew rapidly during the first 10 h of fermentation, reaching 8.06 log CFU/mL. At the end of 14 h fermentation, the viable count was 7.92 log CFU/mL. The results showed that GMDZD, as a substrate for lactic acid fermentation, may be beneficial for the growth of LAB at concentrations consistently higher than the minimum required to sustain a healthy life (7.0 log CFU/mL; Peng et al., 2021). PH is one of the important parameters determination of lactic acid fermentation progress and end (Chen et al., 2018). As shown in Figure 1B, during the fermentation process, pH significantly decreased and titratable acidity significantly increased; pH significantly decreased from 7.01 ± 0.06 to 4.05 ± 0.02, while titratable acidity significantly increased from 4.67 ± 1.54 to 25.33 ± 2.31 g/L. The changes in pH and titratable acidity may be due to the increased concentration of organic acids produced by LAB during fermentation, especially lactic acid production (Yang et al., 2018).

LAB decompose sugars through fermentation to form organic acids. These organic acids are important secondary carbon sources for the proliferation of many microorganisms during food fermentation (Chen et al., 2018). As shown in Figure 1C, the lactic, citric, and acetic acid contents increased significantly after fermentation. Lactic acid is the most abundant organic acid formed after fermentation, increased significantly from 514.23 ± 2.49 μg/mL to 2803.53 ± 44.46 μg/mL, which was 5.46 times higher than that before fermentation. The content of citric acid increased significantly from 232.70 ± 2.43 μg/mL to 338.90 ± 7.18 μg/mL. Acetic acid content increased significantly from 503.47 ± 2.88 μg/mL to 590.83 ± 5.83 μg/mL. After fermentation, malic acid, tartaric acid, and oxalic acid showed fluctuating decreases, and the malic acid content decreased significantly from 385.44 ± 1.μg/mL to 17.19 ± 0.31 μg/mL. Lactic acid content increased significantly after fermentation, and malic acid content decreased significantly. LAB degraded malic acid to lactic acid in the fermentation process, leading to the increase of lactic acid content, namely the malolactic fermentation (MLF). Thus, MLF can be used to decrease juice sourness. The decrease in tartaric acid may be due to the tartaric dehydratase enzyme that enables LAB to convert tartaric acid to oxaloacetic acid and then to lactic acid, acetic acid, and CO_2_ (Landete et al., 2009).

As shown in Figure 1D, the total sugar and reducing sugar content showed the same trend. The total sugar and reducing sugar contents gradually decreased with the extension of fermentation time, and the total sugar content significantly decreased from 79.63 ± 2.29 mg/mL to 61.23 ± 0.61 mg/mL. The reducing sugar content significantly decreased from 14.46 ± 0.36 mg/mL to 12.49 ± 0.16 mg/mL. The reduction of sugar during fermentation was a result of bioconversion into organic acids, and also the result of the proliferation and utilization of LAB (Rakin et al., 2007).

### 3.2. Total phenol content and total flavone content

**Figure 2:**
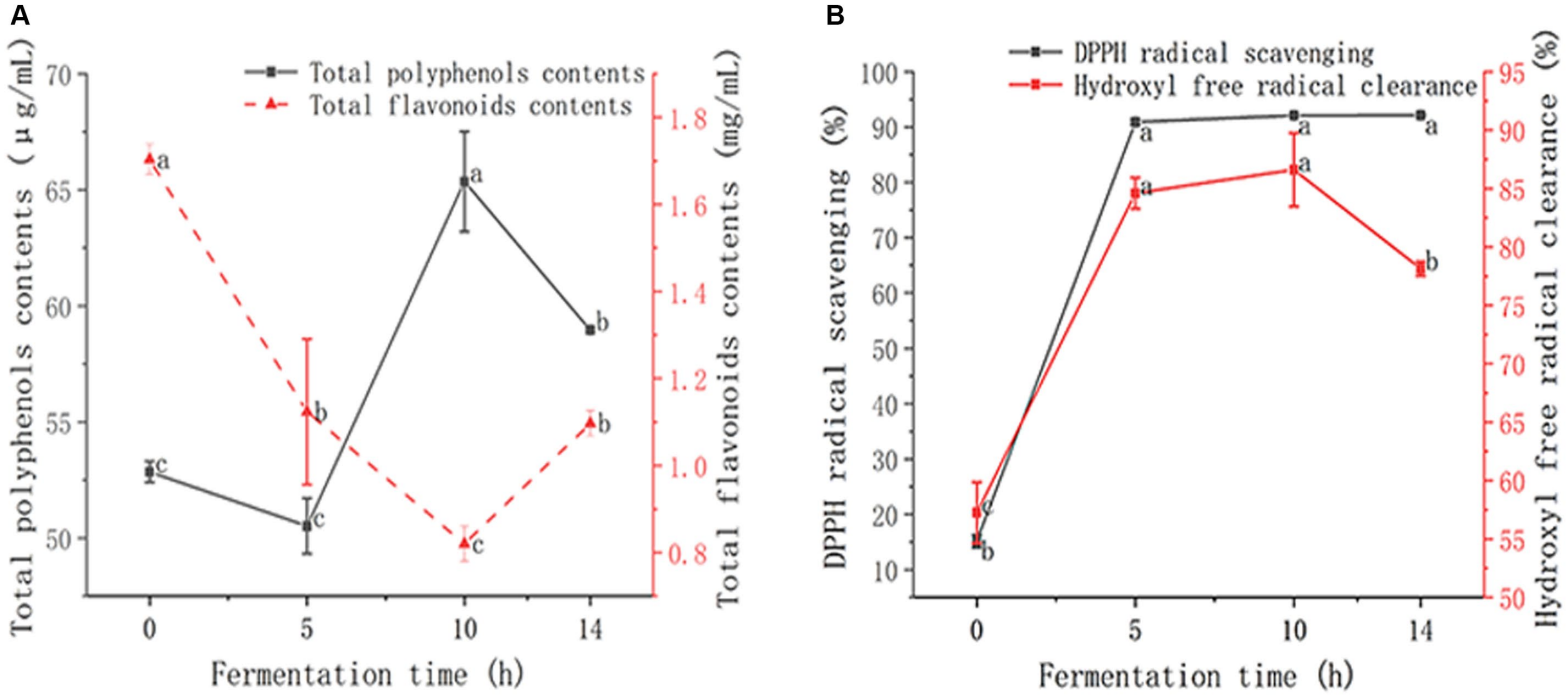
Changes in GMDZD during 14  h of fermentation. The change of TPC and TFC **(A)**, DPPH radical scavenging activity and hydroxyl free radical clearance, the results are expressed as free radical clearance **(B)**. Data are expressed as average (n = 3) ± standard deviation.

As shown in Figure 2A, the TPC showed a fluctuating increasing trend, and the overall trend increased from 52.85 ± 0.45 μg/mL to 65.36 ± 2.16 μg/mL in 0–10 h, and then decreased slightly. TPC at the end of the fermentation was 58.96 ± 0.10 μg/mL, 11.56% higher after fermentation than before fermentation. The increase in TPC after LAB fermentation may be due to the hydrolyze complex phenolic compounds into simpler forms by the hydrolytic enzymes of LAB (Vivek et al., 2019). The TFC showed a trend of fluctuating decline, and decreased from 1.70 ± 0.04 mg/mL to 0.82 ± 0.04 mg/mL at 0–10 h, and then increased at 10–14 h. At the end of fermentation, TFC was 1.10 ± 0.03 mg/mL. The reduced TFC can be explained by the high-molecular-weight phenolic compounds of depolymerization by LAB in GMDZD (Li et al., 2018). TPC and TFC changes were caused by the formation or degradation of phenolic compounds, which are in charge of antioxidant capacity (de Oliveira et al., 2020). It has been proven that LAB strains could produce enzymes to break down cell walls of plant tissue to release bioactive compounds. Feng et al., (2016) fermented pear juice, and during the fermentation process, the TPC increased significantly, and the TFC decreased significantly (Wang et al., 2022). Li et al., (2021) fermented Muzao and Hetian jujube with four kinds of LAB. TPC increased and TFC decreased at the end of fermentation for 48 h (Li et al., 2021), which is consistent with this study’s results.

### 3.3. Changes in antioxidant activities during fermentation

DPPH free radical clearance and hydroxyl free radical clearance are often used to determine the antioxidant capacity of biological materials. Figure 2B shows that the DPPH free radical scavenging rate showed a gradually rising trend direction, increasing significantly from 15.15 ± 1.17% to 90.90 ± 0.41% at 0–5 h, and rising slowly at 5–14 h. At the end of fermentation, the DPPH free radical scavenging rate was 92.14 ± 0.20%, which was 6.08 times higher after fermentation than before. The increase in DPPH•-SA suggests that lactic acid fermentation could increase the compounds with characteristics of proton-donating availability (Kwaw et al., 2018). The hydroxyl free radical clearance showed a fluctuating upward trend, rising rapidly from 57.25 ± 2.61% to 84.60 ± 1.34% at 0–5 h, rising slowly at 5–10 h, and then decreasing slightly. At the end of fermentation, the hydroxyl free radical clearance was 78.14 ± 0.62%, which was 36.49% higher after fermentation than before. They act as reducing agents, free radical scavengers, and singlet oxygen quenchers, phenolic compounds have significant antioxidant activities, which are mainly attributed to the hydrogen atoms transfer or electron donation to free radicals (Verón et al., 2019). After fermentation, the scavenging rate of DPPH and hydroxyl free radicals increases significantly, and the enhancement of antioxidant activity appears to be dependent on an increase in phenols (Ng et al., 2011). These results indicate that the LAB had positive effects on the antioxidant activity of GMDZD.

### 3.4. Variations in γ-aminobutyric acid

As shown in Table 1, the content of GABA showed a fluctuating change, and gradually decreased from 1057.06 ± 5.40 μg/mL to 608.79 ± 2.46 μg/mL at 0–10 h, and increased at 10–14 h. The content of GABA was 1184.50 ± 2.81 μg/mL at the end of fermentation (*p* < 0.05), which was 12.06% higher than that before fermentation; many studies have demonstrated that microbial fermentation can be used to conflate GABA. *Lactobacillus paracasei* catalyzes the decarboxylation of glutamic acid to produce GABA, which may be due to fermentation that produces more free glutamic acid; a portion of the glutamic acid is decarboxylated and transformed into GABA by the catalysis of glutamic acid decarboxylase, causing its content to increase (Xu et al., 2021). Lee et al. fermented sea tangle solution with *L. brevis* BJ20 for 5 days. The content of GABA showed a fluctuating upward trend, which first increased, then decreased, and then increased. At the end of fermentation, the content of GABA increased significantly (Lee et al., 2010). It is similar to the results of this study.

**Table 1:** γ-aminobutyric acid count.

Fermentation time (h)	γ-aminobutyric acid content (μg/mL)
0	1057.06 ± 5.40^b^
5	755.78 ± 3.08^c^
10	608.79 ± 2.46^d^
14	1184.50 ± 2.81^a^

Each value in the table is the mean ± standard deviation (*n* = 3). Means in the same column with different superscript letters indicate significant differences (*P* < 0.05).

### 3.5. LC–MS

#### 3.5.1. Principal component analysis

**Figure 3:**
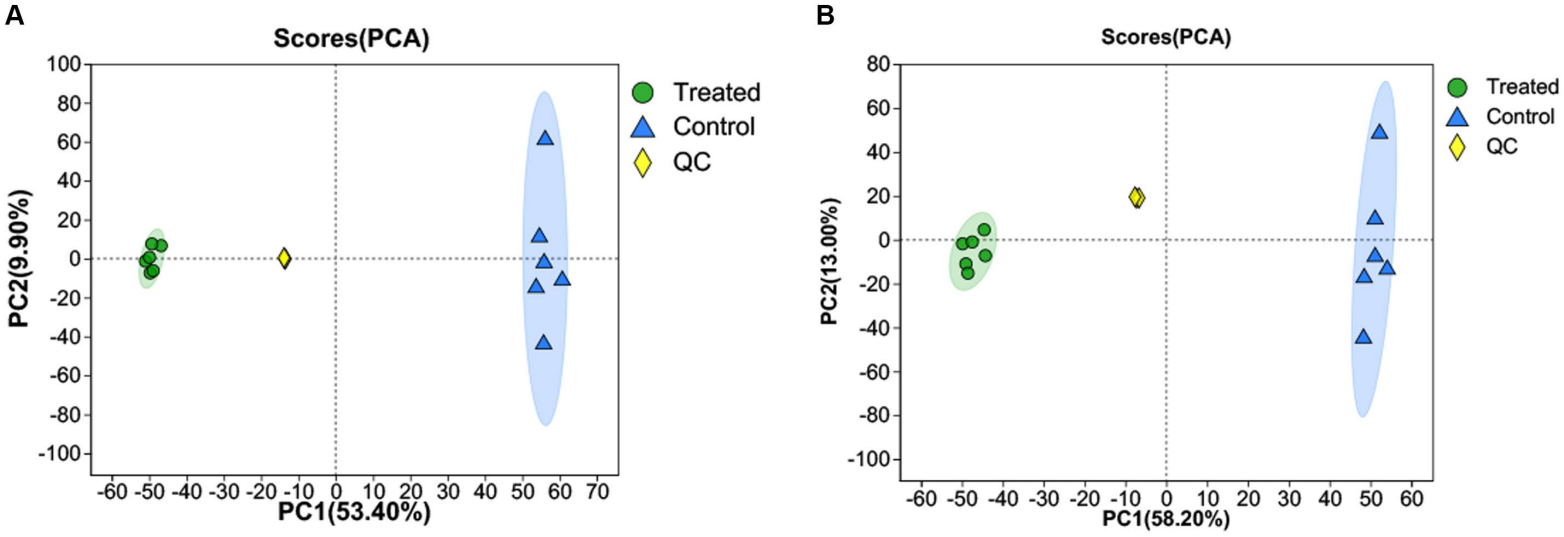
PCA score chart of samples in the positive ion mode **(A)** and negative ion mode **(B)**.

PCA was used to determine the differences in metabolites between GMDZD and fermentation. Figures 3A,B show significant differences in metabolites between treated and control. The two main components take up 63.3 and 71.2% of the total variation, separately (53.4 and 9.90% for PC1 and PC2 in the positive ion mode, and 58.2 and 13% for PC1 and PC2 in the negative ion mode, respectively). The Treated sample on the left side of PC2 is separated from the control sample on the right side. The separation between samples was significant, indicating that the samples were significantly different.

#### 3.5.2. Untargeted metabolomic analysis

**Figure 4:**
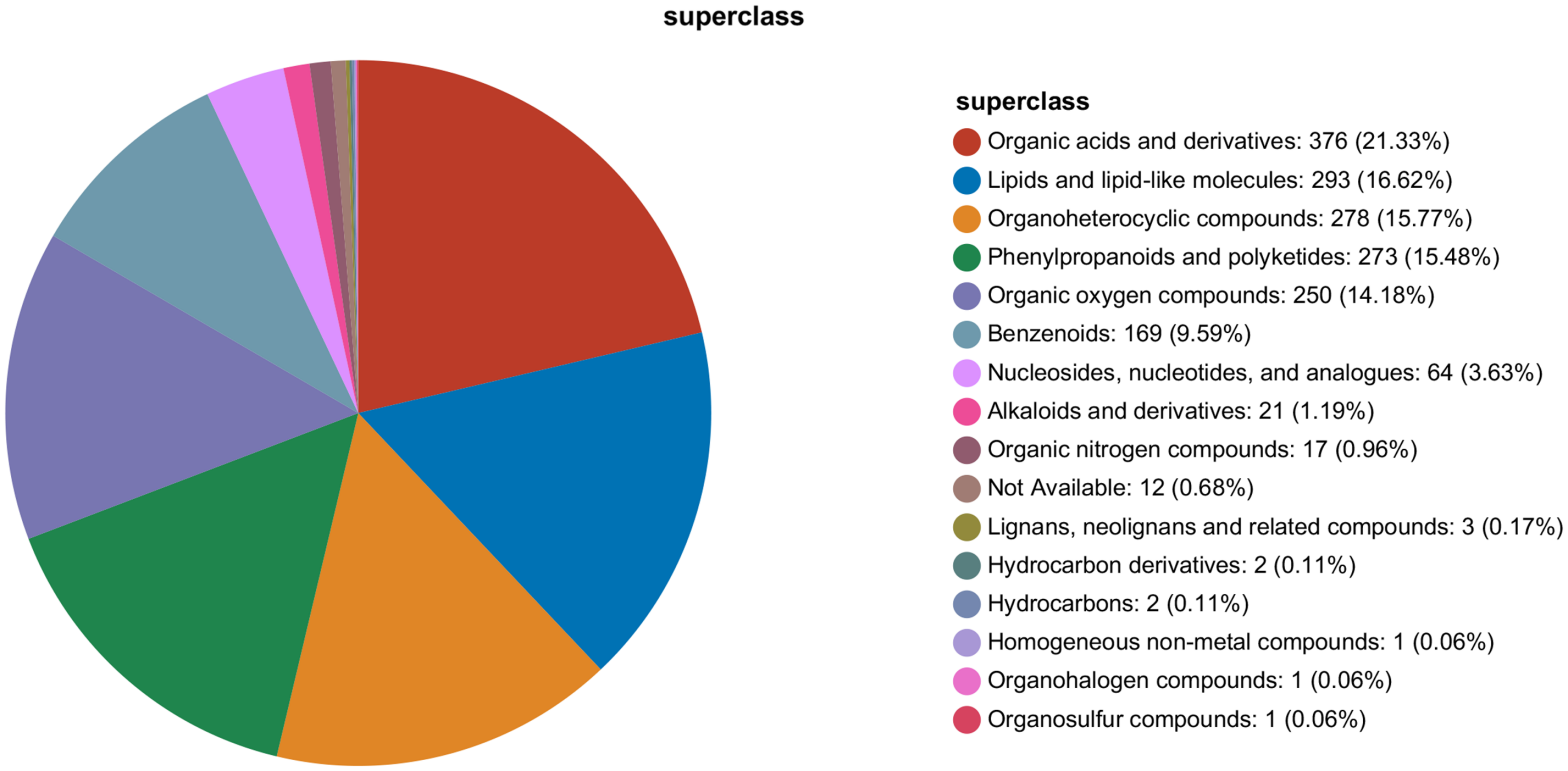
Metabolite classification map.

As shown in Figure 4, UHPLC-QExactive HF-X identified 1763 metabolites, that could be divided into 16 categories. It mainly included I organic acids and derivatives, II lipids and lipid-like molecules, III organoheterocyclic compounds, IV phenylpropanoids and polyketides, V organic oxygen compounds, VI benzenoids, VII nucleosides, nucleotides, and analogues, VIII alkaloids and derivatives, IX organic nitrogen compounds, X not available, XI lignans, neolignans and related compounds, XII hydrocarbon derivatives, XIII hydrocarbons, XIV homogeneous non-metal compounds, XV organohalogen compounds, and XVI organosulfur compounds.

**Figure 5:**
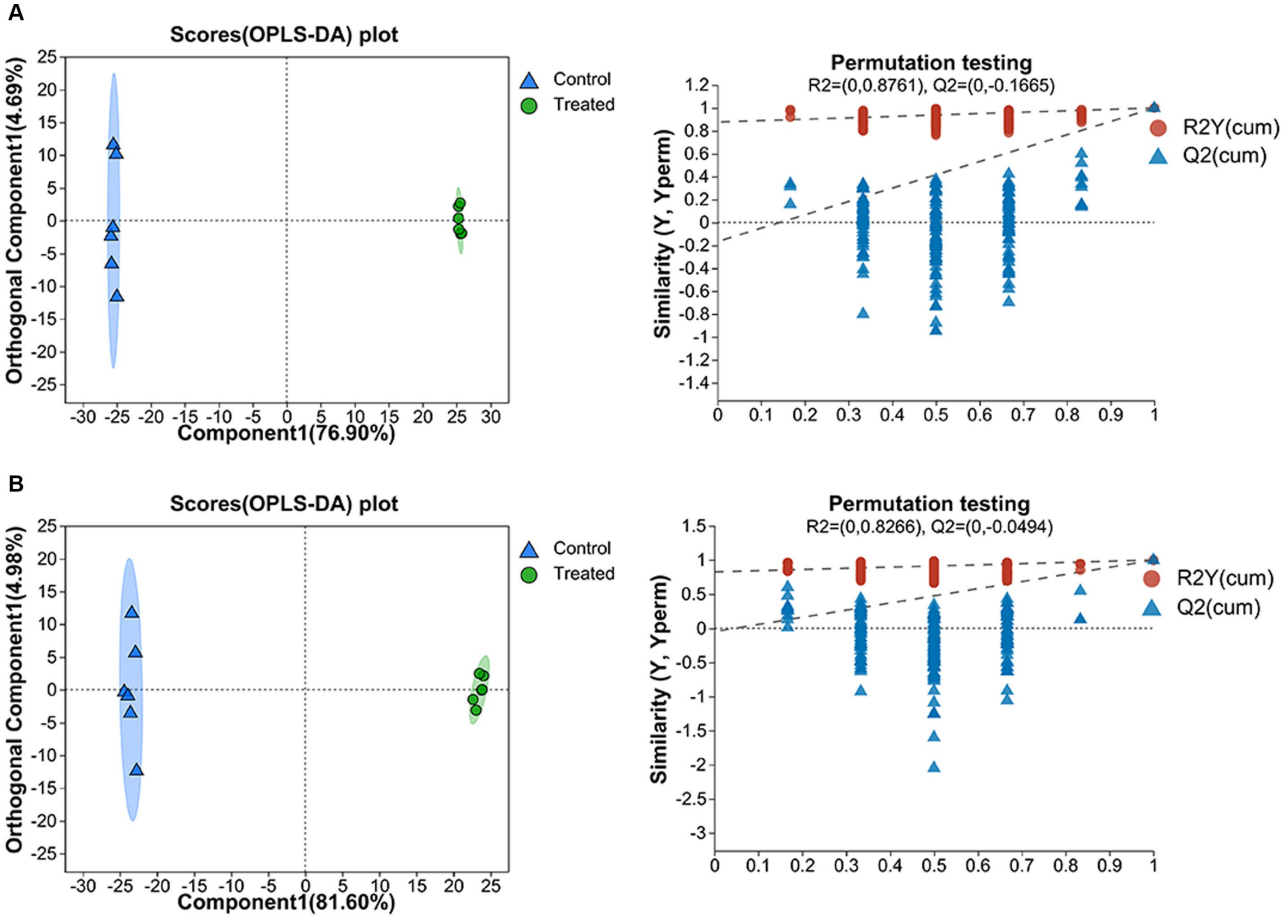
On the left is the OPLS-DA score chart, and on the right is the OPLS-DA model validation in the positive ion mode **(A)** and in the negative ion mode **(B)**.

OPLS-DA was used for research the treated and control metabolites. The score chart and replacement test results are shown in Figures 5A,B, which are similar to those of PCA. The cumulative discriminative explanatory ability of the model established in positive ion mode is *R^2^X* (*cum*) =0.816, *R^2^Y* (*cum*) =1, the forecast capacity of the model *Q^2^* = 0.998, and the cumulative discriminative explanatory ability of the model established in negative ion mode is *R^2^X* (*cum*) =0.866, *R^2^Y* (*cum*) =0.999. And *Q^2^* = 0.998 for model forecast capacity. The closer the three indicators are to 1, the model is more stable, more reliable, and the better the model fits. The above results showed that the OPLS-DA model was reliable and stable and could be used to further recognize the differences in metabolites in the GMDZD before and after fermentation.

**Figure 6:**
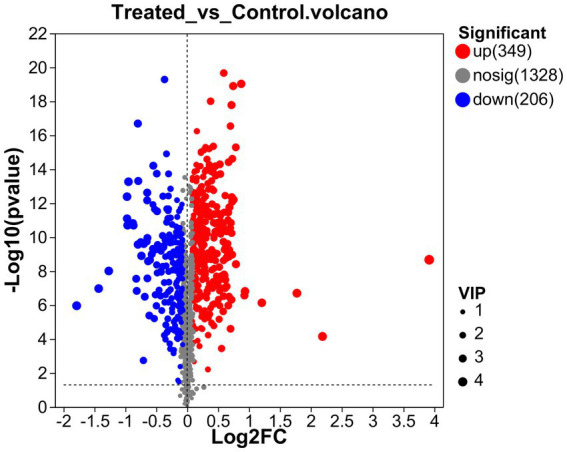
Volcano plot.

**Figure 7:**
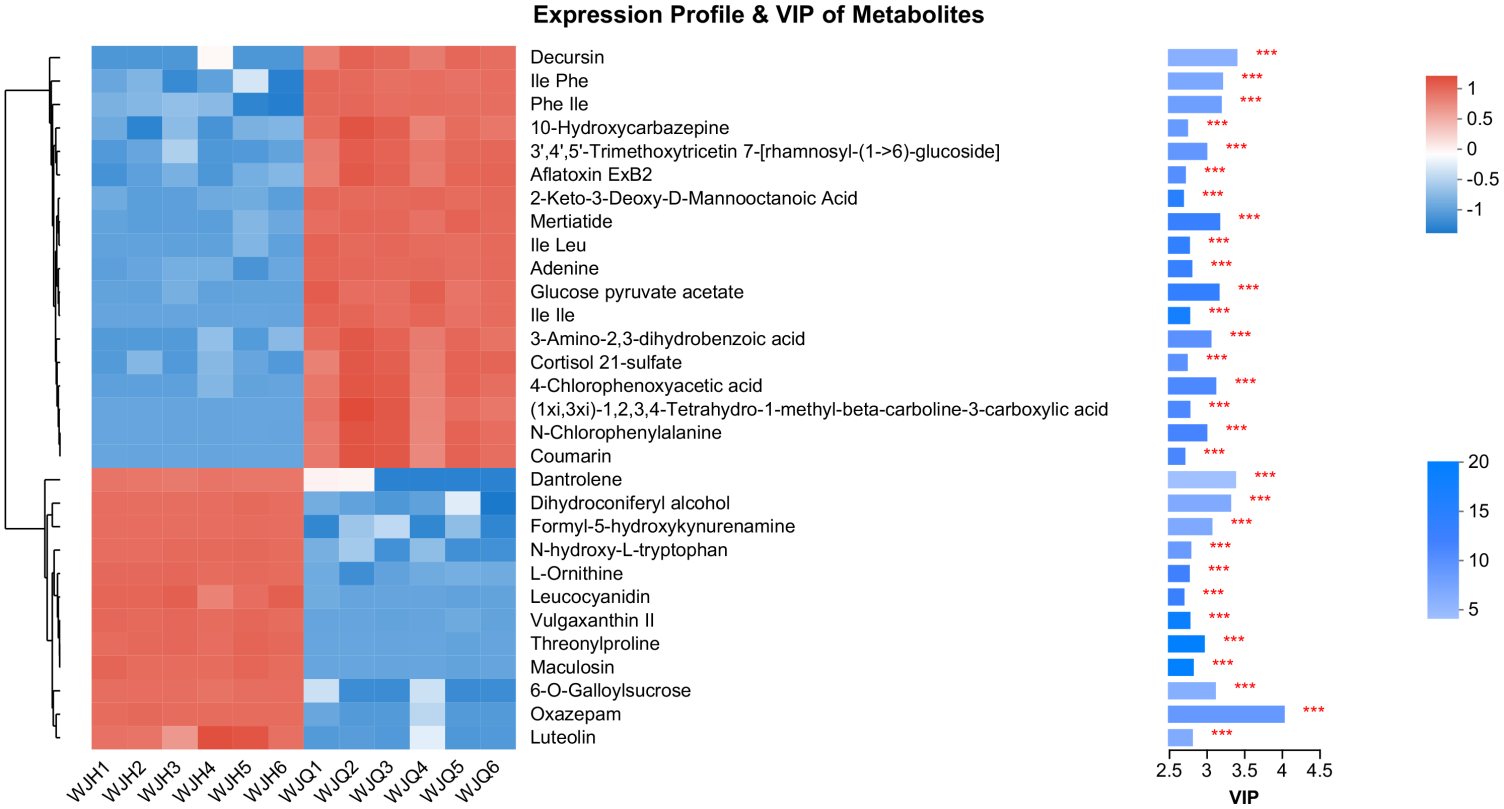
On the left is the metabolite clustering tree diagram, on the right is the metabolite VIP bar chart.

**Figure 8:**
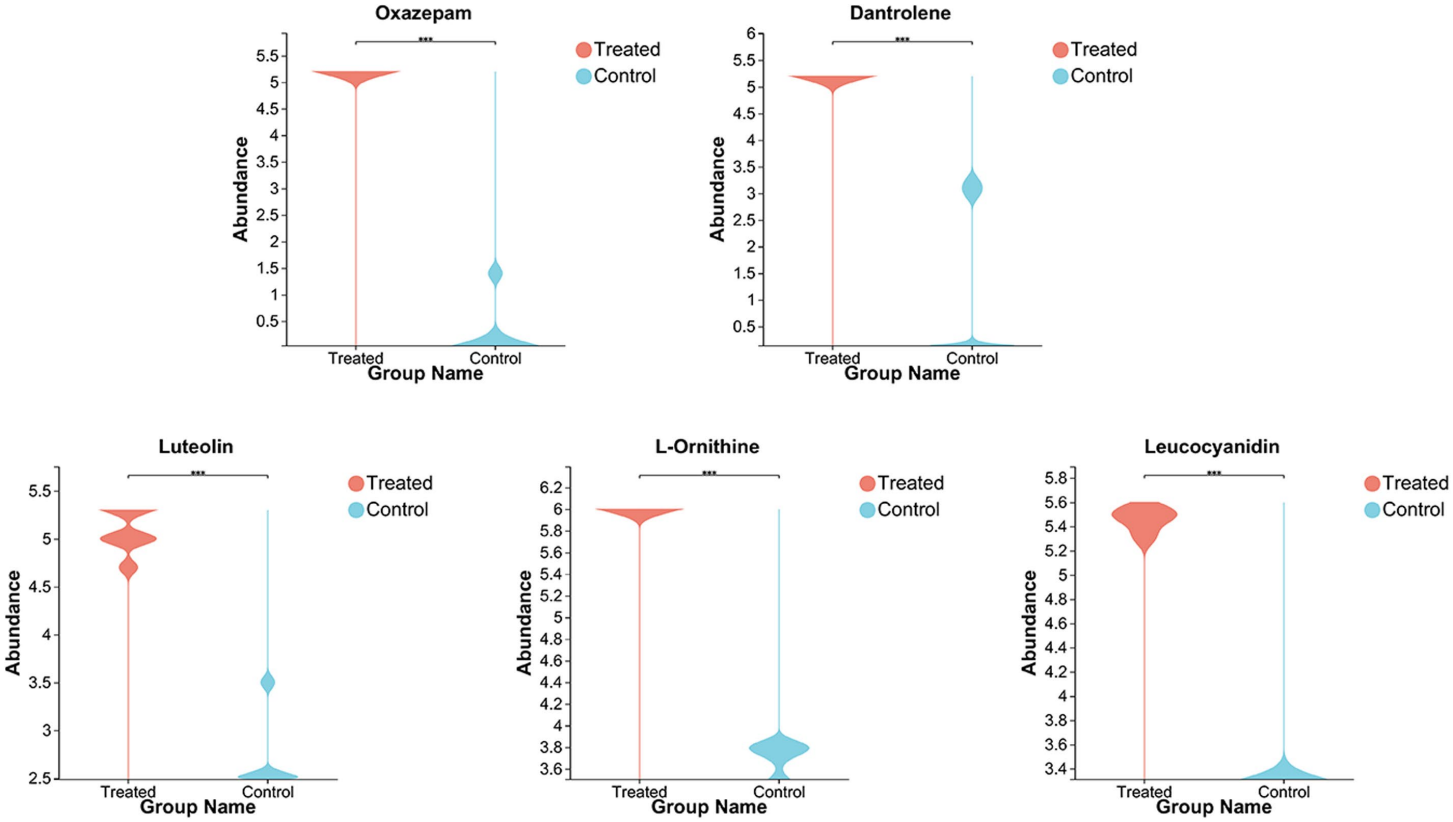
Violin chart of changes in the expression levels of functional substances (including: Oxazepam, Dantrolene, Luteolin, L-Ornithine, and Leucocyanidin).

To better understand the difference in metabolism differences between the Treated (WJH 1–6) and Control (WJQ 1–6) groups, the differential metabolites between Treated and Control were screened based on VIP values >1, *p* < 0.05, and fold-change (FC) > 1 or < 1. Has identified 553 species of positive ion and negative ion mode difference of metabolites, as shown in Figure 6, of which 349 were upregulated and 204 were down regulated; 553 differential metabolites have been identified by the HMDB database, and 522 were identified by consensus. The top 30 differential metabolites with VIP values were selected, as shown in Figure 7. A cluster heat map and VIP bar chart were used to analyze differential metabolites. Metabolites in the top 30 with VIP value differences, the content of five substances increased significantly: oxazepam (FC 15.0953), dantrolene (FC 4.5605), luteolin (FC 1.9007), L-Ornithine (FC 1.6061) and leucocyanidin (FC 1.6483; Supplementary Table S1). Specific changes in their expression levels are shown in Figure 8.

The concentrations of oxazepam and dantrolene significantly increased after fermentation; oxazepam belongs to the benzodiazepines (BZD) class of drugs, and BZD act on the CNS by enhancing the effect of GABA_A_ receptors. They are also used as sedatives, hypnotics, and anxiolytics (Tvete et al., 2016). Dantrolene is metabolized to acetylaminodantrolene, which is formed by the reduction of dantrolene to aminodantrolene and its following acetylation (Amano et al., 2018). Shi et al. demonstrated that intranasal dantrolene is a feasible treatment for Alzheimer’s disease and other dementias (Shi et al., 2019). Luteolin is a natural dietary flavonoid, which usually exists in the form of glycosylation in fruits, vegetables, and herbs. It is provided with antimutagenic, antitumorigenic, antioxidant, and anti-inflammatory features; it may participate in regulating blood glucose levels of health (Park et al., 2009). L-ornithine, a non-protein amino acid and an essential component of the urea cycle, is extensively used to therapy problems of the liver and trauma (Zhang et al., 2017). Kurata et al. (2011) study indicated that oral administration of L-ornithine possibly has a useful influence on brain function associated with anxiety-like behavior. Furthermore, the intracerebroventricular injection of L-ornithine has been proven to lead to calm sedative and hypnotic effects in neonatal chicks under acute stress conditions (Suenaga et al., 2008); L-ornithine also encourages in mice non-rapid eye movement (NREM) sleep (Omori et al., 2011) and improves the quality of human sleep (Miyake et al., 2014). Leucocyanidin is a colorless compound belonging to the colorless anthocyanin subgroup of flavonoids. The anti-inflammatory action and other related health advantages of the fruit can be attributed to its high anthocyanin content (Zhang et al., 2018). Apart from these five substances, the content of formyl-5-hydroxykynurenamine (FC 1.9129) increased significantly after fermentation. Formyl-5-hydroxykynurenamine was a metabolite of 5-hydroxytryptamine, a monoamine transporter in the hypothalamus, increased significantly after fermentation (Liu et al., 2013). Maculosin (FC 1.6752) content increases significantly after fermentation and due to its powerful antioxidant and non-toxic characteristics, it is probable a leading candidate drug for various cosmetic and therapeutic applications (Paudel et al., 2021).

In addition to the metabolites with the top 30 VIP values among the differential metabolites, the contents of epigallocatechin gallate (EGCG; FC 1.6325), epicatechin (FC 1.4706), epigallocatechin (FC 1.5216), and other substances were significantly increased, all of which belong to phenylpropane and polyketones and exist in the form of tea polyphenols. They exhibit antioxidant, anti-inflammatory, and antitumor effects (Kuo et al., 2014). EGCG is the main polyphenol in green tea. One study found that EGCG was effective in attenuating fatigue (Leggio and Addolorato, 2009). The experiment found that the content of gallic acid (FC 1.0308) in GMDZD increased remarkably, owing to after fermentation, epicatechin was used up by LAB and produced gallic acid with strong antioxidant activity. In LAB fermentation processes besides malic and lactic acids, succinic acid (FC 1.0273) is a usual metabolite. During fermentation, LAB can transform malic acid into succinic acid under anaerobic conditions. At the same time, a portion of succinic acid is obtained from the amino acids decomposed by some LAB (Ji et al., 2023).

In summary, the contents of luteolin, leucocyanidin, EGCG, gallic acid, and other substances increased significantly after fermentation, and they all had antioxidant and anti-inflammatory effects. The contents of L-ornithine, oxazepam, dantrolene increased significantly after fermentation, and they all had the effects of promoting sleep and relieving anxiety. The significant increase in the content of these substances mentioned above indicates that the LAB fermentation of GMDZD has a positive effect. After LAB fermentation, the function and bioavailability of GMDZD can be improved.

## 4. Conclusion

In this study, the fermentation of GMDZD with LAB significantly improved its TPC, antioxidant activity, and GABA content. LC–MS non-targeted metabolomics was used for the first time to determine the changes in small and medium molecular substances in the GMDZD before and after fermentation. Among the top 30 differential metabolites with VIP values, five functional metabolites were up regulated, namely Oxazepam, Dantrolene, Luteolin, L-ornithine, and Leucocyanidin, which have sedative, sleep-promoting, and antioxidant effects. The content of active substances with antioxidant, anti-inflammatory, and sleep-promoting effects increased significantly after the fermentation of GMDZD, indicating that LAB fermentation significantly improved the function of GMDZD, and laid a theoretical foundation for its industrial production.

## Data availability statement

The raw data supporting the conclusions of this article will be made available by the authors, without undue reservation.

## Author contributions

LW: Conceptualization, Data curation, Investigation, Methodology, Software, Writing – original draft, Writing – review & editing. YL: Conceptualization, Data curation, Investigation, Methodology, Software, Writing – original draft, Writing – review & editing. ZH: Writing – review & editing. ZZ: Writing – review & editing. LZ: Conceptualization, Funding acquisition, Supervision, Writing – review & editing. YX: Conceptualization, Funding acquisition, Supervision, Writing – review & editing. HY, SX, and SL: Software, Writing – review & editing.

## Footnotes

^1^http://bioconductor.Org/packages/release/bioc/html/ropls.Html

^2^https://cloud.majorbio.com
