# Supplementary material for: Fermentation improves antioxidant capacity and γ-aminobutyric acid content of Ganmai Dazao Decoction by lactic acid bacteria
Source: Front Microbiol. 2023 Nov 2;14:1274353. doi: 10.3389/fmicb.2023.1274353 (PMC10652878; doi:10.3389/fmicb.2023.1274353)
Supplement: Supplementary file 1 [file Table_1.docx]

| Metabolite name | RT | Mass error | Molecular formula | Fragmentation Score | Theoretical fragmentation Score | M/Z |
| --- | --- | --- | --- | --- | --- | --- |
| Dantrolene | 2.9887 | -2.18934570437162 | C14H10N405 | - | 47.1 | 359.062634711 |
| L-Ornithine | 0.5044 | -0.0263496315672785 | C5H12N202 | 75.8 | - | 133.097150585 |
| Luteolin | 3.8795 | -1.43795925580048 | C15H1006 | - | 56.9 | 319.080817893 |
| Oxazepam | 0.5044 | -4.14101620983429 | C15H11CIN202 | 49.3 | - | 287.056997192 |
| Leucocyanidin | 2.2146 | -0.818599653239824 | C15H1407 | - | 36.4 | 307.080978666 |
| Formyl-5-hydroxykynurenamine | 2.0980 | -1.4476327200614099 | C10H12N203 | - | 38.2 | 226.118316553 |
| Maculosin | 2.7561 | -1.77354424568276 | C14H16N203 | 74.5 | - | 261.122907486 |
| Epigallocatechin gallate(EGCG) | 3.6294 | -1.29598092859461 | C22H18011 | - | 47.7 | 459.091594155 |
| Epicatechin | 2.8730 | -0.8682051589641919 | C15H1406 | 73.3 | - | 291.08606275 |
| Epigallocatechin | 2.8296 | 0.737518050563823 | C15H1407 | 84.9 | - | 305.066902089 |
| Gallic acid | 2.0503 | -3.27654638912852 | C7H605 | 94.4 | - | 169.013689775 |
| Succinic acid | 0.8678 | -7.35104267590058 | C4H604 | 79.9 | - | 117.01846462 |
